# Integron Gene Cassettes and Degradation of Compounds Associated with Industrial Waste: The Case of the Sydney Tar Ponds

**DOI:** 10.1371/journal.pone.0005276

**Published:** 2009-04-23

**Authors:** Jeremy E. Koenig, Christine Sharp, Marlena Dlutek, Bruce Curtis, Michael Joss, Yan Boucher, W. Ford Doolittle

**Affiliations:** 1 Department of Biochemistry and Molecular Biology, Dalhousie University, Halifax, Nova Scotia, Canada; 2 Department of Biological Sciences, Macquarie University, Sydney, Australia; 3 Department of Civil and Environmental Engineering, Massachusetts Institute of Technology, Cambridge, Massachusetts, United States of America; University of Wisconsin-Milwaukee, United States of America

## Abstract

Integrons are genetic platforms that accelerate lateral gene transfer (LGT) among bacteria. They were first detected on plasmids bearing single and multiple drug resistance determinants in human pathogens, and it is abundantly clear that integrons have played a major role in the evolution of this public health menace. Similar genetic elements can be found in nonpathogenic environmental bacteria and in metagenomic environmental DNA samples, and it is reasonable to suppose that integrons have facilitated microbial adaptation through LGT in niches outside infectious disease wards. Here we show that a heavily impacted estuary, exposed for almost a century to products of coal and steel industries, has developed a rich and unique cassette metagenome, containing genes likely to aid in the catabolism of compounds associated with industrial waste found there. In addition, we report that the most abundant cassette recovered in this study is one that encodes a putative LysR protein. This autoregulatory transcriptional regulator is known to activate transcription of linked target genes or unlinked regulons encoding diverse functions including chlorocatechol and dichlorophenol catabolism. Finally, only class 1 integrase genes were amplified in this study despite using different primer sets, and it may be that the cassettes present in the Tar Ponds will prove to be associated with class 1 integrase genes. Nevertheless, our cassette library provides a snapshot of a complex evolutionary process involving integron-meditated LGT likely to be important in natural bioremediation.

## Introduction

Integrons are genetic platforms for lateral gene transfer (LGT) that facilitate adaptation in bacteria, first recognized and named by Ruth Hall and Hatch Stokes in 1989 [1]. These evolved systems comprise two components, the first encoding a single site-specific tyrosine recombinase (integrase) that effects integration and excision of one or more elements of the second type, gene cassettes (Figure S1). Recombination most commonly targets an integron-associated attachment site (*attI*) immediately adjacent (upstream) to the integrase gene (*intI*) and a site, *attC* (also referred to as 59-be), found within individual mobile circular gene cassettes [2]. Integrated cassettes (as many as 200 in the case of some integron arrays on *Vibrio* chromosomes [3]) are thus linearized, and separated by recombinant *attC* sites. These arrays are dynamic insofar as closely related organisms show drastically different profiles of cassettes [4] which typically encode promoterless open reading frames (ORFs) mostly oriented in the same direction and thought to be transcribed from an integron-associated common promoter (P_c_) after integration [5].

Before the late 1990s, most characterized integrons were hospital-derived, transposon-associated and plasmid-borne, carrying between zero and half-a-dozen representatives of a repertoire of about 100 antibiotic-resistance determining cassettes adjacent to a “class 1” *intI* gene (one of several homologous integrase sequence types) [6], [7]. Genomic sequencing, especially of *Vibrio* species and other proteobacteria, has since revealed much larger chromosome-located cassette arrays, with ORFs of various function [6]. Such other functions are largely unknown, however, and specialized environments comparable to infectious disease wards in which specialized cassette reservoirs might also have evolved need to be examined for accumulation of cassettes with niche-specific function. Indeed, it has been reported that integrons appear to be abundant in contaminated sites [8] and environments that have long been exposed to anthropogenic pollutants might harbour integrons enriched with cassette-encoded enzymes that promote their degradation.

Nova Scotia provides an ideal test site for this latter conjecture: the Sydney Tar Ponds. Steel production began at this urban Cape Breton location in the early 1900s and continued for more than 80 years. In that time at least 700,000 tonnes of toxic by-products, including total petroleum hydrocarbons (TPHs), benzene, toluene, ethyl-benzene, xylene (BTEX), polycyclic aromatic hydrocarbons (PAHs) and heavy metals from smelting and associated steel production processes were deposited into a tidal estuary that empties into Sydney Harbour and ultimately the Atlantic Ocean [9]. The early 1980s marked the beginning of environmental site assessments [10], and further analyses revealed that there is significant groundwater contamination as well as deposition of several million tonnes of contaminated particulate matter within the industrial site and the surrounding community [11]. Furthermore, human health research in Sydney has revealed an increase in cancer incidence, mortality [12], [13] and, congenital anomalies compared to the rest of Nova Scotia and Canada [14] (see Figure S2 for a timeline of events associated with steel production in Sydney).

Although the site is now slated to be solidified and stabilized, we were able to obtain Tar Pond samples prior to encapsulation and identify microbial community members using standard 16S rRNA-based phylotyping methods, and to derive some idea of the richness of the pool of gene cassettes that has accumulated there using an environmental polymerase chain reaction (PCR) or specifically, cassette PCR. This entails use of degenerate primers targeted against known *attC* sequences in the polymerase chain reaction applied to an environmental DNA sample, in this case a shallow sediment sample from the south Tar Pond. Cassette PCR has shown its effectiveness in recovering single and multiple cassettes (including interspersed *attC* sites) in the hands of Stokes and colleagues [15] and others [16], and by us in an analysis of cassette diversity from sites in and around Halifax Harbour [17].

## Results and Discussion

### Bacterial and archaeal diversity in the Sydney Tar Ponds

The taxonomic distribution of ribotypes (phylotypes) recovered in this study is listed in Table S1. Sixty-seven of 181 16S rRNA gene sequences amplified with universal bacterial primers were identified as unclassified *Methanosarcina,* as were 164 of 199 sequences recovered with archaeal-specific primers. Organisms from this group are known to perform acetoclastic methanogenesis, dismuting acetate to carbon dioxide and methane (CH_3_COOH→CO_2_+CH_4_) [18]. Indeed, gas chromatography analysis of the gas phase emitted from Tar Pond sediment supports *in situ* methanogenesis (data not shown). Methanogenesis by acetoclastic methanogens is known to occur in anaerobic zones near the source of contaminants [19]. This process is mediated by *Syntrophus aciditrophicus* [20] and indeed we recovered five unclassified Syntrophus ribotypes (Table S1). Furthermore, acetate is thought to be produced by organisms that can anaerobically oxidize many contaminants using electron acceptors such as sulfate, nitrate and iron(III) [19]. Organisms that are known to decontaminate using these alternative electron acceptors include *Geobacter* species, and *Desulfobacterium* species as well as *Dehalococcoides* species. Three ribotypes that identify these organisms were recovered in this study (Table S1 in bold-type).

Sequences sharing 97% nucleotide identity or more were assigned to the same Operational Taxonomic Unit (OTU), and the total richness of 16S rRNA genes in the Sydney Tar Ponds (number of OTUs) was calculated using the program DOTUR [21]. This resulted in an estimated ∼152 and ∼80 OTUs for 16S rRNA genes amplified with the bacterial universal and archaeal-specific primers, respectively (Figure S3). This value is low considering that the environment was originally a marine estuary and would be expected to harbour a ten-fold higher ribotype-richness (a recent study estimates bacterial and archaeal ribotype richness in marine sediments to range from 2000 to 3000 OTUs) [22]. Reduced bacterial and archaeal diversity might reflect the selective regime imposed by the contamination of this site.

### *IntI* diversity in the Sydney Tar Ponds

Integrase diversity was assessed by PCR using both class 1-specific and degenerate universal integron integrase (*intI*) primers [23]. All *intI* sequences obtained with either primer set were of class 1, hitherto most frequently associated with antibiotic resistance (Figure S4). It may be the case that the cassettes present in the Tar Ponds will prove to be associated with class 1 integrase genes as no other types of integron integrase were found by us despite using different primer sets. Of course it may also be that there are additional *intI* genes that we did not sample because they are too divergent and so would not be amplified by either primer set.

### Gene cassette diversity in the Sydney Tar Ponds

A total of 708 gene cassette sequences were obtained, of which ∼22% (160) potentially encode proteins with known homologs, consistent with other environmental surveys of integron cassettes [1], [16], [23], [24]. The Chao1 richness index [25] was calculated and it was found that there are liklely more than 1900 cassette-types (nucleotide sequence similarity >70%) in the Tar Pond library of gene cassettes thus illustrating the abundance of genes belonging to the mobile gene pool at this site. The functional distribution of these inferred proteins is summarized in Table S2. The most abundant of the cassettes with an ascribable function recovered in this study (41 or ∼26% of those with identifiable functions) is one that encodes a homolog to the LysR regulator, or more specifically, the DNA-binding domain of this regulator.

The LysR family comprises more than 50 members of autoregulatory transcriptional regulators (LTTRs) and these are found in a diverse range of bacteria [26]. LTTRs are known to activate divergent transcription of linked target genes or unlinked regulons encoding extremely diverse functions including chlorocatechol and dichlorophenol catabolism in *Pseudomonas putida* [27], [28]. The cassette-encoded protein identified in this study has a 97 percent amino acid identity to the DNA binding domain of the LysR regulator in *Pseudomonas fluorescens* Pf-5 [29]. In this organism, LysR is physically linked to genes involved in cell division and cell cycle, phosphate metabolism as well as peripheral pathways for catabolism of aromatic compounds. Without specific genetic assays, it is difficult to say for certain whether these cassette-encoded LysR LTTRs are promoting the expression of gene cassettes in integrons or affecting the expression of other genes in the host genome. Nevertheless, it seems likely that genome-incorporation of cassettes such as these will have an affect on the phenotype of the organism.

**Figure 1:**
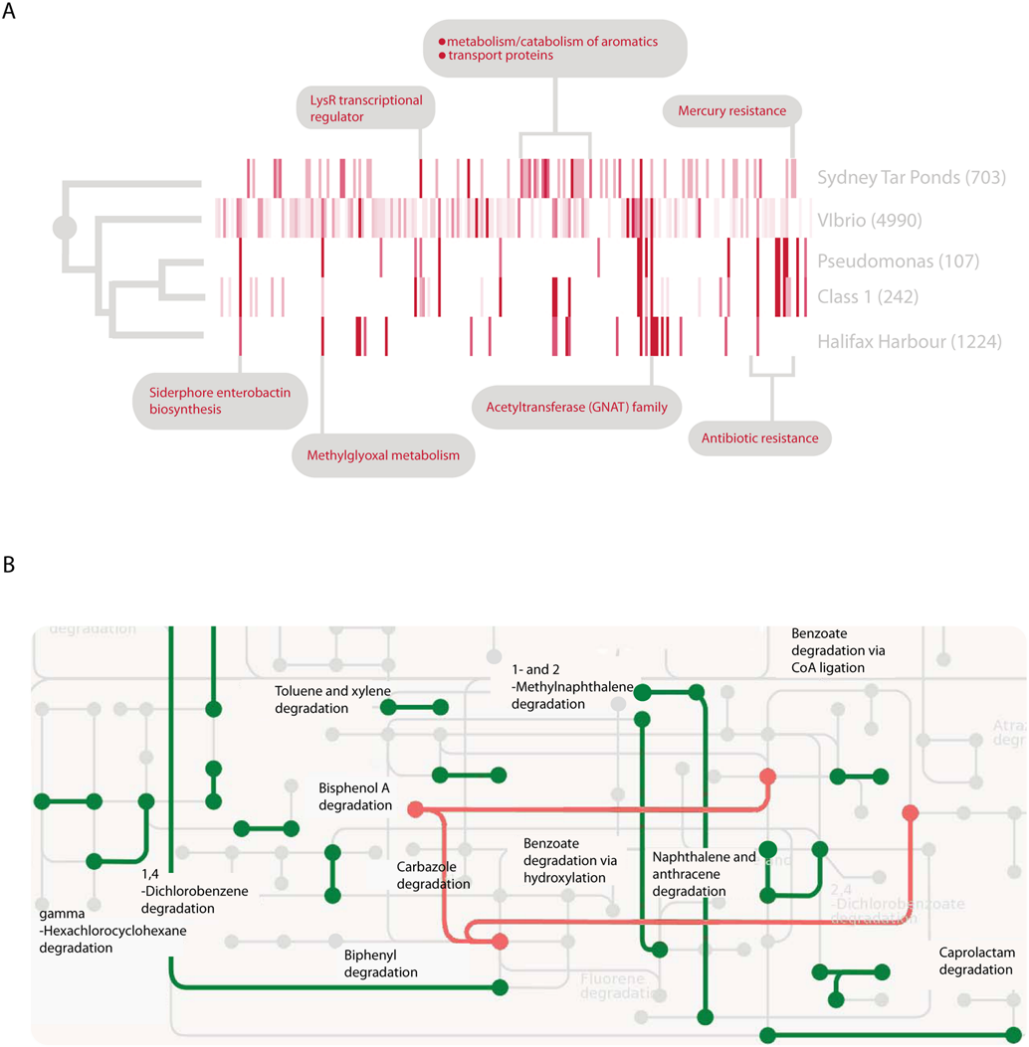
Integron gene cassette-encoded function. (A) Comparative functional analysis of different integron gene cassette pools. Cassette-encoded proteins were functionally described into role categories using MG-RAST and these data were used to construct normalized heat maps also using MG-RAST [30]. The intensity of the red bars indicates relative abundance of that particular functional role category in each gene pool. The numbers in parentheses represent the size of each sampled gene pool and the grey circle represents a P-value of less than 0.0001 that these differences might be attributed to chance. (B) Metabolic atlas of integron gene cassettes from the cassette metagenome and those amplified from the Sydney Tar Ponds. Green lines represent the cassette-encoded metabolic potential found in the Tar Ponds while the red lines indicate the functional overlap of cassette sequences from the Tar Ponds as well as the entire cassette metagenome.

As we had conjectured, a number of cassettes (22, or ∼15% of those with identifiable functions) have inferred functional roles in the degradation of contaminants present in the Sydney Tar Ponds (Table S3) and these are summarized in Table S4. Among these are three cassettes encoding the phenylacetic acid degradation protein PaaI, two benzoate transport proteins, two periplasmic molybdenum-binding (ModA) proteins, in addition to a cassette encoded 4-carboxymuconolactone decarboxylase, mercuric ion reductase and, catechol 1,4-dioxygenase. The majority of such functions have not before been found associated with the mobile gene pool consisting of integron gene cassettes. Using a heat map approach (Figure 1A), we compared the functional distribution of integron gene cassettes amplified from the Tar Ponds to other well-sampled integron gene cassette pools. These included cassettes from the *Vibrio* and *Pseudomonas* genera, the cassette pool known to facilitate drug-resistance (referred to as class 1 associated cassettes) as well as cassettes from another well-sampled environment (Halifax Harbour sewage outfall). By this measure, it is apparent that the functional profile of cassettes obtained from the Tar Ponds is quite different from any other sampled integron gene cassette pool (Figure 1A). This claim is supported by the *r*×*c* contingency test performed on the functional distribution of all known gene cassettes *versus* those amplified from the Sydney Tar Ponds. We observe that these two distributions are significantly different from one another with a P-value of less than 0.0001.

The metabolic atlas of functionally described cassettes highlights the biochemical reactions that cassette-encoded proteins from the Tar Ponds might facilitate, as compared to the entire cassette metagenome previously known (Figure 1B). This analysis was performed by first annotating cassette data with MG-RAST [30] and then using the atlas visualization algorithm developed by the Kyoto Encyclopedia of Genes and Genomes (KEGG) [31]. This analysis illustrates that integron gene cassettes amplified from the Tar Ponds potentially encode more biochemical reactions involved in the degradation of compounds associated with industrial processes than those found collectively in the ∼7000 cassettes sampled from other environments studied to date.

The cassette-encoded catabolic enzymes that potentially degrade compounds associated with industrial processes like catechol 1,4-dioxygenase (EC 1.13.11.1) and 4-carboxymuconolactone decarboxylase (EC 4.1.1.44) are downstream reactions, suggesting that these enzymes likely have a role in degrading catabolic intermediates produced by other enzymes found in contaminated sites. Indeed, it has been suggested that although a complete pathway for a particular substrate may not exist in a single organism, partial and complementary pathway segments may exist in different organisms [32]. Therefore, we do not suggest that bioremediation of sites like this one is carried out by integrons alone, rather, integrons are likely involved in the acquisition of new genes that may augment the fitness of a bacterial host that finds itself among a plethora of contaminants and their catabolic intermediates. Furthermore, since integrons are LGT agents, it may be that they are building clusters of genes in an operon-like structure, an evolutionary strategy proposed by Lawrence and Roth [33]. Accordingly, the cassettes we observe presently could be part of an intermediate stage in the evolution of integrons that are moving toward specialized catabolic arrays.

**Figure 2:**
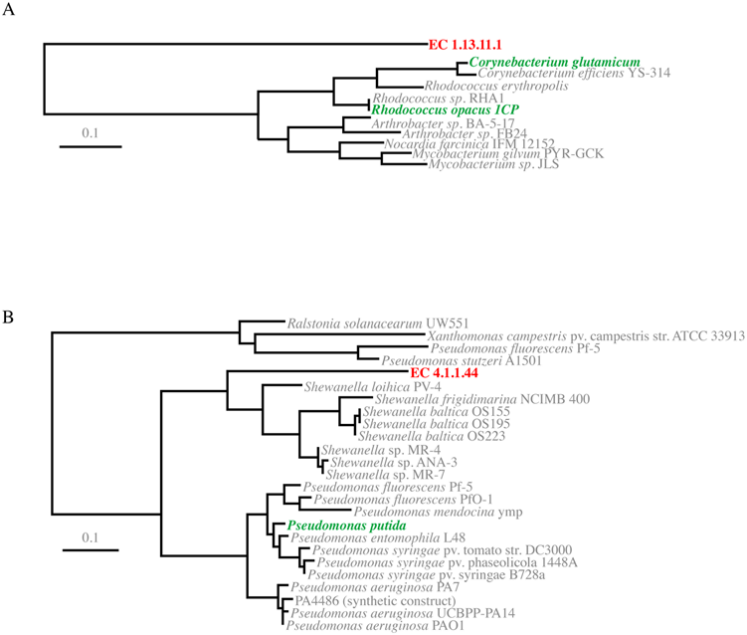
Phylogenetic analysis of cassette-encoded homologs of enzymes amplified from the Sydney Tar Ponds and known to be involved in the degradation of industrial pollutants. (A) PHYML analysis of integron gene cassette-encoded putative catechol 1,4-dioxygenase (1.13.11.1), a protein implicated in benzoate as well as 2,4-dichlorobenzene degradation. (B) PHYML analysis of integron gene cassette-encoded putative 4-carboxymuconolactone decarboxylase (EC 4.1.1.44) a protein implicated in benzoate degradation. All nodes in both trees have bootstrap values of 85 or higher. Genes that have been functionally characterized are illustrated in green [51], [52], [53].

Several of these gene cassette-encoded enzymes are particularly suggestive in terms of their potential to facilitate bioremediation. Specifically, putative catechol 1,4-dioxygenase (EC 1.13.11.1), an enzyme implicated in 1,4-dichlorobenzene degradation as well as benzoate degradation, a degradation pathway that is downstream to a number of catabolic intermediates present in the Tar Ponds and one that would facilitate funneling these molecules to other biological processes such as phenylalanine biosynthesis and oxidative phosphorylation. Amino acid phylogeny of this cassette-encoded enzyme indicates that while this gene is an out-group to the sampled catechol 1,4-dioxygenases, it is nevertheless most closely related to organisms known to catabolize aromatics (Figure 2A). In addition, cassette-encoded 4-carboxymuconolactone decarboxylase (EC 4.1.1.44) also implicated in benzoate degradation, was identified in our dataset and this enzyme groups with homologs from *Shewanella,* a genus that has generated much interest in the field of bioremediation given its ability to use a diversity of electron acceptors (Figure 2B).

Five of the catabolic enzymes associated with the degradation of industrial contaminants (EC 1.14.13.-, EC 4.1.3.39, EC 4.2.1.17, EC 1.1.1.-, EC 2.3.1.-) are also implicated in other conserved biological functions (Table S5). Yet given the study site, we suggest that these genes, likely mobilized via gene cassettes, may provide their host(s) with duplicates of these catabolic enzymes. Therefore, these genes may evolve more freely to better suit their new role in bioremediation, which would be consistent with previous suggestions that extant enzymes can be modified for processes for which they were not originally evolved to perform [34]. Indeed there is evidence for metabolic repertoire expansion in bacteria made possible through processes such as duplication of catabolic genes [35].

### Long-walk PCR of cassette-encoded EC 4.1.1.44

Any PCR approach is subject to false positives, especially when degenerate primers are used, as was the case in this study. Therefore, in order to confirm that one putative contaminant degrading enzyme encoded within a cassette, 4-carboxymuconolactone decarboxylase (EC 4.1.1.44) is part of a cassette array, we used the long walk PCR protocol developed by Katz and colleagues 1999 [36]. Sequence data generated by this technique revealed that this enzyme was indeed part of a cassette array. We observed three additional cassettes, two of them encoding novel hypothetical proteins, and one encoding a conserved hypothetical protein. All of these ORFs were flanked by the integron attachment site *attC* in addition to being oriented in the same direction, which is most typical of gene cassette arrays (Figure S5). In addition to these, we identified eighteen cassette products encoding *attC* recombination sequences that would not amplify with the primers designed by Stokes and colleagues [15] (Figure S6).

### Taxonomic assignment of gene cassettes

**Figure 3:**
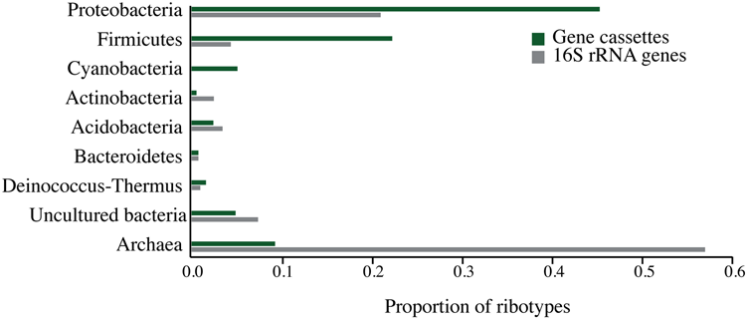
Taxonomic distribution of integron gene cassettes amplified from the Sydney Tar Ponds compared to the sample site's 16S rRNA gene profile.

We used the software package MEGAN, an algorithm based on best BLAST scores [37] to analyze the taxonomic distribution of gene cassettes amplified from the Tar Ponds (Figure 3). In total 189 of 708 cassettes could be assigned using this method. Of these, 158 belonged to the Proteobacteria, the most abundant phylum observed in this 16S rRNA gene library (Table S1). Of these cassettes, 61 were identified as originating from *Pseudomonas*, the most abundant genus and one implicated in contaminant degradation. Figure 3 also includes the profile of 16S rRNA genes amplified from the Tar Ponds and although the principal inhabitants of the site sampled are methanogenic archaea, very few cassettes were taxonomically assigned to the Archaea.

### Culture-dependent analysis of integrons

**Figure 4:**
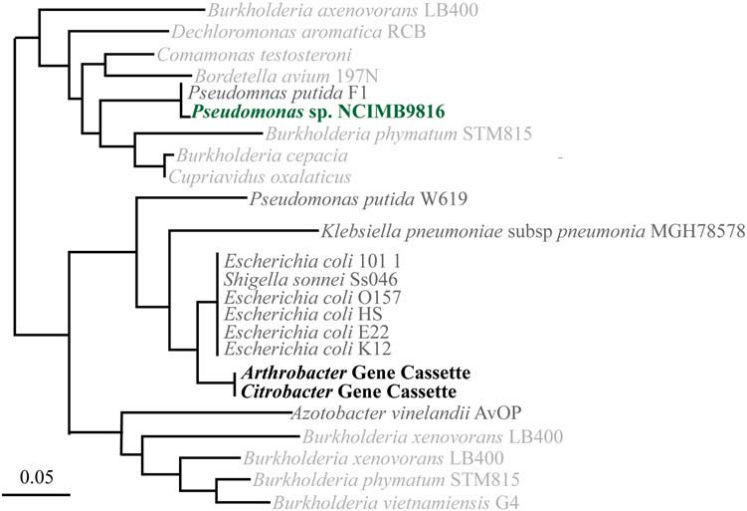
Phylogenetic analysis for cassette-encoded 4-hydroxy-2-oxovalerate aldolase (EC 4.1.3.39) amplified from putative *Arthrobacter* sp. and *Citrobacter* sp. All nodes have bootstrap support of 85 or higher and sequences that have been functionally characterized are coloured green [54]. Taxonomic assignment of each gene is indicated in light grey (Betaproteobacteria) or dark gray (Gammaproteobacteria). Gene cassette sequences recovered from isolates in this study are in bold-type.

Twenty bacterial isolates were cultured from the Sydney Tar Ponds and screened for the presence of integron integrase genes and gene cassettes by PCR. Two bacterial isolates where found to encode integrons by this screen; a putative *Arthrobacter* sp. and a *Citrobacter* sp. Both of these isolates are known to be contaminant degraders [34], [35], [38] and they contain integrons, judged by the presence of an *intI* gene, in addition to several cassettes amplified and sequenced from both organisms. The integron integrase genes of both isolates grouped with the class 1 integron clade illustrated in Figure S4. The cassettes amplified from these isolates, with their ascribed functions, are listed in Table S6. Interestingly, these isolates share a number of cassettes, among these a cassette encoding 4-hydroxy-2-oxovalerate aldolase (EC 4.1.3.39), an enzyme involved in the catabolism of aromatics. Indeed, this enzyme is implicated in catabolic pathways leading to the degradation of industrial pollutants including benzoate degradation via hydroxylation, biphenyl degradation, toluene and xylene degradation, 1,4-dichlorobenzene degradation, fluorene degradation, carbazole degradation, and styrene degradation (Table S5). Specifically, this enzyme catalyzes the cleavage of 4-hydroxy-2-oxovalerate (a downstream product in all pathways mentioned above) to acetaldehyde+pyruvate. Phylogenetic analysis of these amino acid sequences suggests a between-phylum LGT (Figure 4). While these cassettes appear to be closely related to genes from *Escherichia coli*, organisms that have not demonstrated the capacity to facilitate bioremediation, their role in syntrophic bioremediation processes should not be discounted. Furthermore, genome studies of the *Arthrobacter aurescens* strain TC1 (isolated because of its propensity to catabolize the herbicide atrazine and because it belongs to a genus recognized for its far reaching metabolic potential) revealed that it has likely expanded its metabolic repertoire through processes such as duplication of catabolic genes in addition to funneling metabolic intermediates generated by plasmid-borne genes to chromosomally-encoded pathways [35]. Regardless of their origin the integron seems to be mobilizing cassettes encoding enzymes involved in cleaving ring structures like the ones in PAHs and these appear to be mobilized between disparate lineages of bacteria.

### Concluding remarks

Collecting and examining cassettes from environments in which particular selection pressures might be inferred is a fruitful approach to assessing the functional diversity of the gene cassette metagenome, which may ultimately prove to be enormous. The Sydney Tar Ponds, with almost a century of exposure to a rich mix of compounds representing both challenges and opportunities to its microbiota, offers an ideal test case. Other surveys of similar environments have been limited in scale: a single potentially niche-relevant cassette was described from a heavy-metal-contaminated mine tailings site [24]. In the Tar Ponds we found 22 gene cassettes potentially involved in the catabolism of seven of the fourteen most prevalent industrial pollutants sampled (Table S3 and Table S4). Like the initially discovered and extensively studied antibiotic resistance determinants which first called attention to integrons, the cassettes discovered here may prove to be associated with the class 1 IntIs (no other types of integron integrase were found by us despite using degenerate primers designed in [23]). Although the principal inhabitants of the site sampled are methanogenic archaea, no archaeal integrons have been previously described, and our MEGAN analysis (Figure 3) suggest that the gene cassettes we have found are predominantly of proteobacterial origin (although this could be the result of sampling biases of the organisms whose genomes are represented in the database). Almost certainly, phylogenetically disparate organisms are sharing cassettes (Figure 4), but it is also likely that such cassettes have been earlier recruited from chromosomal contexts, since only the sequences in bold-type in Figure 4 are from cassettes. Accumulation of a rich cassette metagenome will likely prove to be a complex and historically contingent process, and further metagenomic investigation of our samples may provide insights into this history as well as the role of LGT processes in bioremediation. For this reason sites such as the Sydney Tar Ponds are not only a public health liability but also a resource for studying the evolutionary processes leading to bioremediation.

## Materials and Methods

### Sample collection

Tar Pond sludge was collected from the shoreline of the south Tar Pond located near Muggah Creek, Cape Breton, NS, Canada (Northing: 5114173, Easting: 0717240), a site rich in contaminants associated with coal and steel industrial processes [39]. Five grams of tar sludge was subjected to bead beating DNA extraction techniques using the BIO 101® Systems FastDNA® Kit (Cat #6540-400) and the purified whole community DNA was used for subsequent molecular techniques.

### *intI* gene amplification from cultured organisms

Five grams of sludge collected from the Tar Ponds were added to 50 mL of sterile water (Fisher Bioreagents, cat no. P561-1), plated on a 2% glucose agar Petri dish [40] and grown at room temperature. Isolates were screened by PCR using class 1 integrase and gene cassette primers as described below.

### PCR amplification

Domain-biased archaeal (A751F and U1406R) and universal bacterial (U515F and U1406R) [41] primers were used to amplify the 16S rRNA gene from the Tar Pond DNA. Reaction mixtures contained 20 µl of Master Mix (Eppendorf cat. no. 954140423), 10 µl of 10 pM primer (forward+reverse), 2 µl of template DNA [25ng/µl] and 18 µl of dH_2_O. This mixture was subjected to the following PCR protocol using a Peltier thermal cycler-200: 94°C for 3 min, (94°C for 30 s, 55°C for 30 s, 68°C for 2 min 30 s)*29 cycles, 68°C for 5 min.

Integron gene cassettes were amplified with degenerate primers targeting conserved *attC* regions described in Stokes *et al.,* 2001 [15]. These primers are: TCSGCTKGARCGAMTTGTTAGVC and GCSGCTKANCTCVRRCGTTAGSC, HS287 and HS286 respectively [15]. Reaction mixtures were amplified with a Peltier thermal cycler-200 using the following program: 94°C for 3 min, (94°C for 30 s, 54°C for 30 s, 68°C for 2.5 min)*35, 68°C for 5 min. Amplified cassettes were subjected to electrophoresis on a 1% agarose gel and products that produced the typical integron gene cassette profile (multiple bands that range in size from 0.3 to 1.5 Kbps) were selected for cloning.

PCR using primers specific for the *intI* gene was performed using class 1-specific primers (HS463A: CTGGATTTCGATCACGGCACG and HS464: ACATGCGTGTAAATCATCGTCG) (Hatch Stokes personal communication) as well as the more degenerate primer set *intI*-255F and *intI*-948R that were designed in Elsaied *et al*., 2007 [23]. These amplification reactions were performed using the same protocol as cassette PCR.

### Long walk PCR amplification

The long walk PCR protocol was followed according to Katz and colleagues [36]. Using nomenclature consistent with that study, we employed the following primers: GSP 1, ACCCTTCTTCTAACGGC; GSP2, TTTCGTGCGGCAAACGAAGTC; and GSP3, TGAATTCATCTCAAATCTCG.

### Clone library construction

Each PCR reaction was independently cloned into the pCR-XL-TOPO vector (Invitrogen cat. no. K4750-10) and transformed into TOP10 chemically competent *E. coli* cells (Invitrogen cat. no. K4750-10). Samples were plated on 1×LB+Kanamycin [50 µg/mL]+X-gal plates [2 mL/L] for blue white colony screening.

### DNA sequencing

Clones were miniprepped by alkaline lysis with GeneMachine robotics (Genomic Solutions). Sequencing of miniprepped clones was performed using the DYEnamic™ ET Dye Terminator Kit (MegaBACE) and run on a MegaBACE™ 1000 (Amersham). Both forward and reverse sequencing reactions were performed, thus providing two sequencing reads for each clone. These sequence data are publicly available under the EMBL accession numbers: FM866473-FM867587.

### Sequence analysis

DNA sequences were trimmed, edited and assembled with PhredPhrap and Consed [42], [43], [44]. 16S rRNA gene sequences were then subjected to the Bellerophon chimera check available on the greengenes 16S rRNA gene database and workbench (http://greengenes.lbl.gov/) [45]. Non-chimeric 16S rRNA genes were aligned against the 16S greengenes rRNA gene database using the NAST aligner [45] and then classified using the NCBI taxonomic nomenclature [45]. Distance matrices to be used in the Distance-Based OTU and Richness determination algorithm (DOTUR) [21] were calculated using the DNAML option of DNADIST (PHYLIP package) available in the greengenes toolbox [45]. Integron gene cassette sequences were functionally annotated using MG-RAST (http://metagenomics.nmpdr.org) built as a modified version of the RAST server [30]. Normalized heat maps were also generated using MG-RAST and the different gene pool arrays were hierarchically clustered using Kendall's tau similarity metric. The Self Organizing Map (SOM) was generated using 20,000 iterations also using the Kendall's tau similarity metric in the freeware Cluster 3.0 (http://www.geo.vu.nl/~huik/cluster.htm). Heat map graphics were generated using JavaTreeView [46]. The program MEGAN [37] was implemented using the default settings to taxonomically assign gene cassettes amplified from the Tar Ponds as well as the Halifax Harbour.

### Compilation of statistics on diversity

The DOTUR algorithm [21] was used to assess both 16S rRNA gene and gene cassette diversity in the Sydney Tar Ponds. The furthest neighbor approach was used to calculate rarefaction data and the Chao1 richness indices at the 97% and 70% nucleotide similarity level of Operational Taxonomic Units (OTUs) for 16S rRNA genes and gene cassettes respectively. When analyzing these estimates with greater scrutiny, by assuming that the OTUs were sampled in the order they were sequenced, it was clear that the estimate continued to grow in the case of the rRNA genes amplified with archaeal-specific primers and the gene cassette samples. Furthermore, we analyzed the range of the 95% CI as a function of sampling effort by using the collector's curve for universal bacterial and archaeal-specific primers as well as gene cassettes as suggested in [21]. There was a slight positive correlation with sequencing effort (R^2^ = 0.27) for the archaeal-specific primer set suggesting that the Chao1 estimate's uncertainty increases with additional sampling and thus the 95% CI is artificially low for sequence data generated with the archaeal-specific primers. This was not the case for the universal primer set, suggesting that we have sequenced a significant number of clones from this library in order to more accurately calculate this Chao1 richness estimate with 95% CI (Figure S7).

### Phylogeny

Protein alignments of translated cassette-encoded genes as well as *intI* sequences and their homologs from the NCBI database were generated using ClustalW [47] and manually edited to remove ambiguous positions. Maximum Likelihood (ML) phylogenetic analyses of cassette-encoded proteins were performed using PHYML (with the WAG amino acid substitution matrix, a rate heterogeneity model with gamma-distributed rates over eight categories, and an alpha parameter estimated from the data) [48]. Bootstrap support values were calculated with the same parameters (100 replicates).

Nucleotide alignments of amplified *intI* genes and a diverse set of class 1 *intI* genes obtained from [49] were also aligned using ClustalW [47] and manually edited. Nucleotide phylogeny was performed using RA×ML [50]. All free model parameters were estimated by RA×ML using the GAMMA+P-Invar model of rate heterogeneity with an ML estimate of the alpha-parameter. Bootstrap support values were calculated with the same parameters (100 replicates).

## Supporting Information

Figure S1 Simplified genetic structure of the integron.
(2.29 MB TIF)

Figure S2 Timeline of events related to steel production in Sydney, Cape Breton, Nova Scotia, Canada.
(9.88 MB TIF)

Figure S3 Rarefaction analysis of 16S rRNA gene clone libraries obtained from the Sydney Tar Ponds. Rarefaction analysis was performed using Distance-based OTU and Richness determination (DOTUR) [21]. Chao1 richness estimates at 97% sequence identity are indicated for each library.
(8.37 MB TIF)

Figure S4 Integron integrase diversity in the Sydney Tar Ponds. (A) General IntI diversity. A diverse selection of IntI protein sequences were retrieved from NCBI for phylogenetic analysis, among these are eight from a deep-sea vent environmental survey of integrons illustrated in bold-type [23]. The number of cassettes in the associated array is indicated in square brackets where data is available. All integrase genes amplified in this study cluster within the black clade that is made up of class 1-associated integrases. Grey circles represent greater than 80% bootstrap support. (B) Nucleotide phylogeny of diverse class 1 *intI* sequences. This analysis includes all *intI* sequences amplified from the Tar Ponds as well as those collected from diverse isolates in addition to sequences amplified in a recent environmental survey of class 1 integrases [49]. RA×ML phylogeny of these sequences resolved three well-supported clades indicated by the bootstrap values at their nodes.
(0.91 MB TIF)

Figure S5 Partial integron gene cassette array obtained by long-walk PCR on DNA extracted from the Sydney Tar Ponds.
(1.95 MB TIF)

Figure S6 Nucleotide alignment of divergent *attC* recombination sequences embedded in multiple-cassette-amplicons. Primers used for cassette PCR are included at the top of the alignment [15]. None of the illustrated *attC* sequences would have been amplified with this primer pair. Asterisks represent positions in the alignment with identical nucleotides. Accession numbers and cassette-encoded functions are listed to the left of the alignment; putative functions are listed relative to their position (left or right) of the given *attC*. The sequence lengths of the *attC*s are indicated to the right.
(0.69 MB TIF)

Figure S7 Dot plot of Chao1 95% CI range obtained from each ordered sampling incidence of 16S rRNA gene sequences amplified by either the archaeal-biased set (A) or the universal primer set (B).
(0.59 MB TIF)

Table S1 (0.03 MB XLS)

Table S2 (0.02 MB XLS)

Table S3 (0.01 MB XLS)

Table S4 (0.02 MB XLS)

Table S5 (0.02 MB XLS)

Table S6 (0.02 MB XLS)
