# Supplementary figures and images for: Integron Gene Cassettes and Degradation of Compounds Associated with Industrial Waste: The Case of the Sydney Tar Ponds
Source: PLoS One. 2009 Apr 23;4(4):e5276. doi: 10.1371/journal.pone.0005276 (PMC2669170; doi:10.1371/journal.pone.0005276)

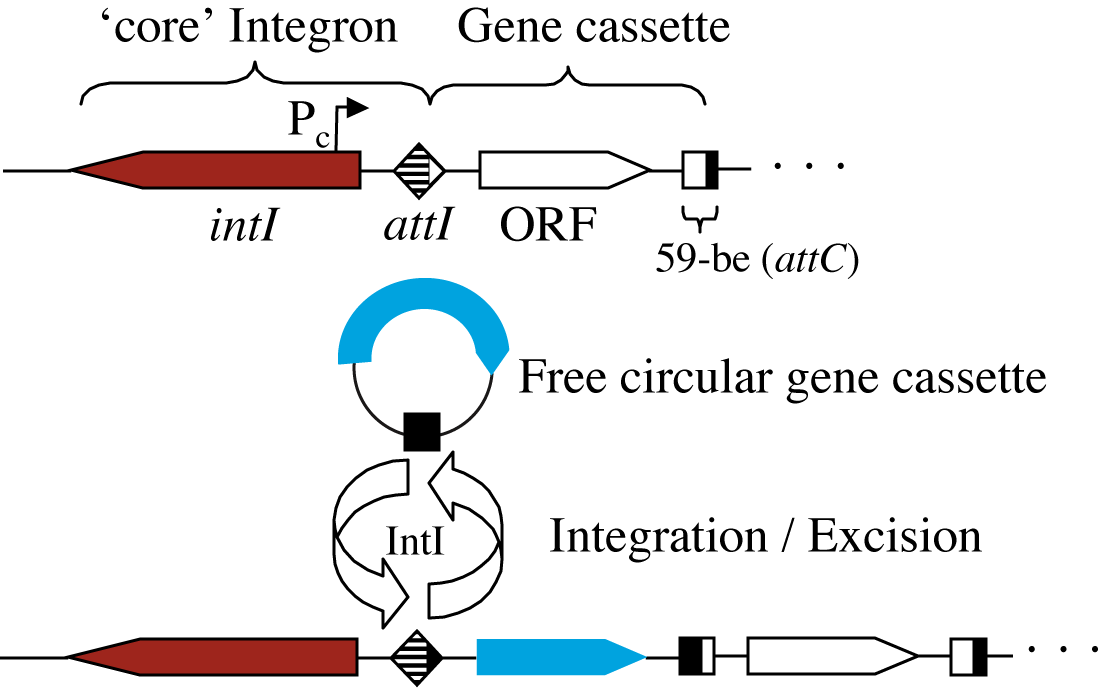

Supplement: Figure S1 — Simplified genetic structure of the integron. (2.29 MB TIF) [file pone.0005276.s001.tif]

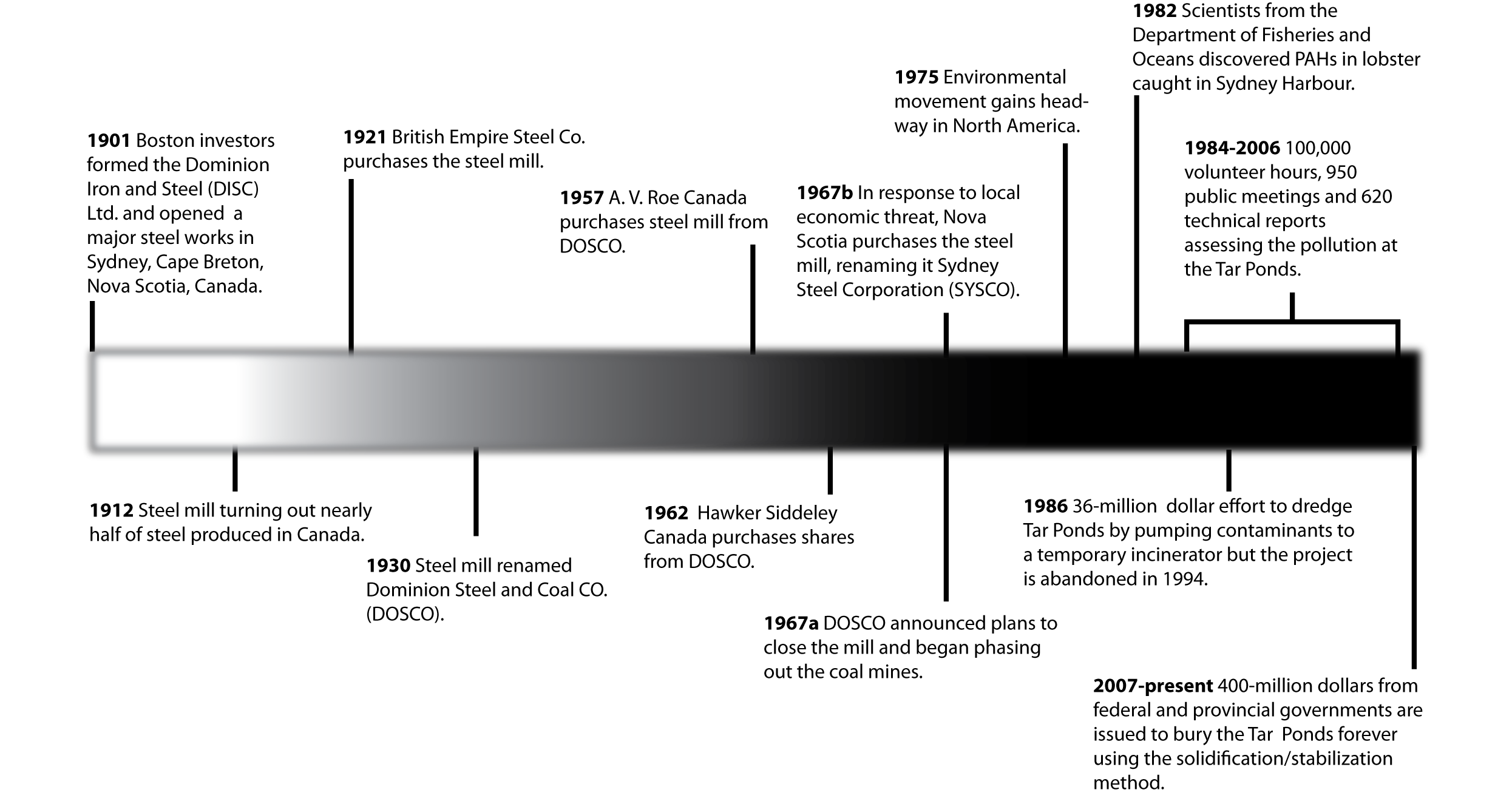

Supplement: Figure S2 — Timeline of events related to steel production in Sydney, Cape Breton, Nova Scotia, Canada. (9.88 MB TIF) [file pone.0005276.s002.tif]

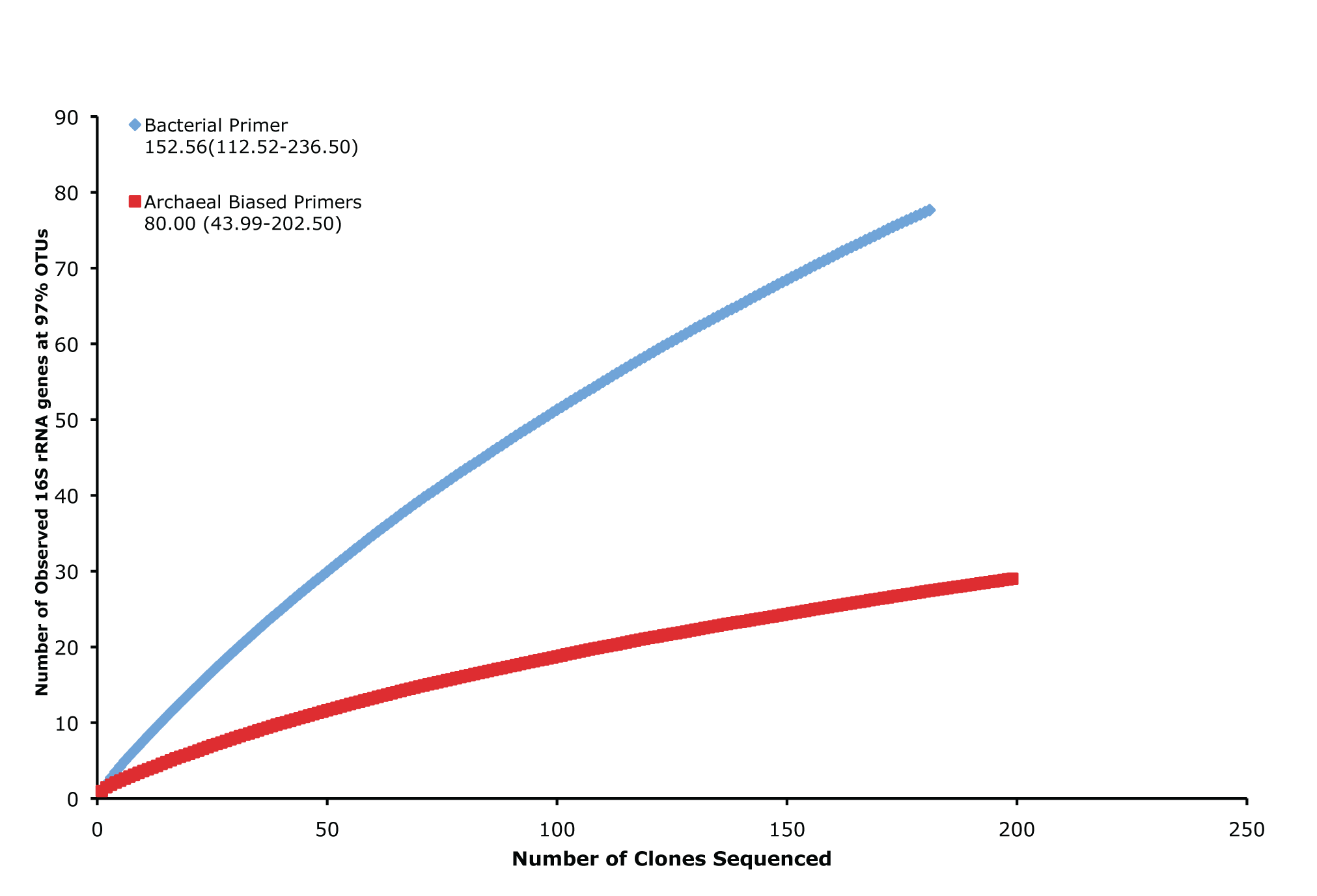

Supplement: Figure S3 — Rarefaction analysis of 16S rRNA gene clone libraries obtained from the Sydney Tar Ponds. Rarefaction analysis was performed using Distance-based OTU and Richness determination (DOTUR) [21]. Chao1 richness estimates at 97% sequence identity are indicated for each library. (8.37 MB TIF) [file pone.0005276.s003.tif]

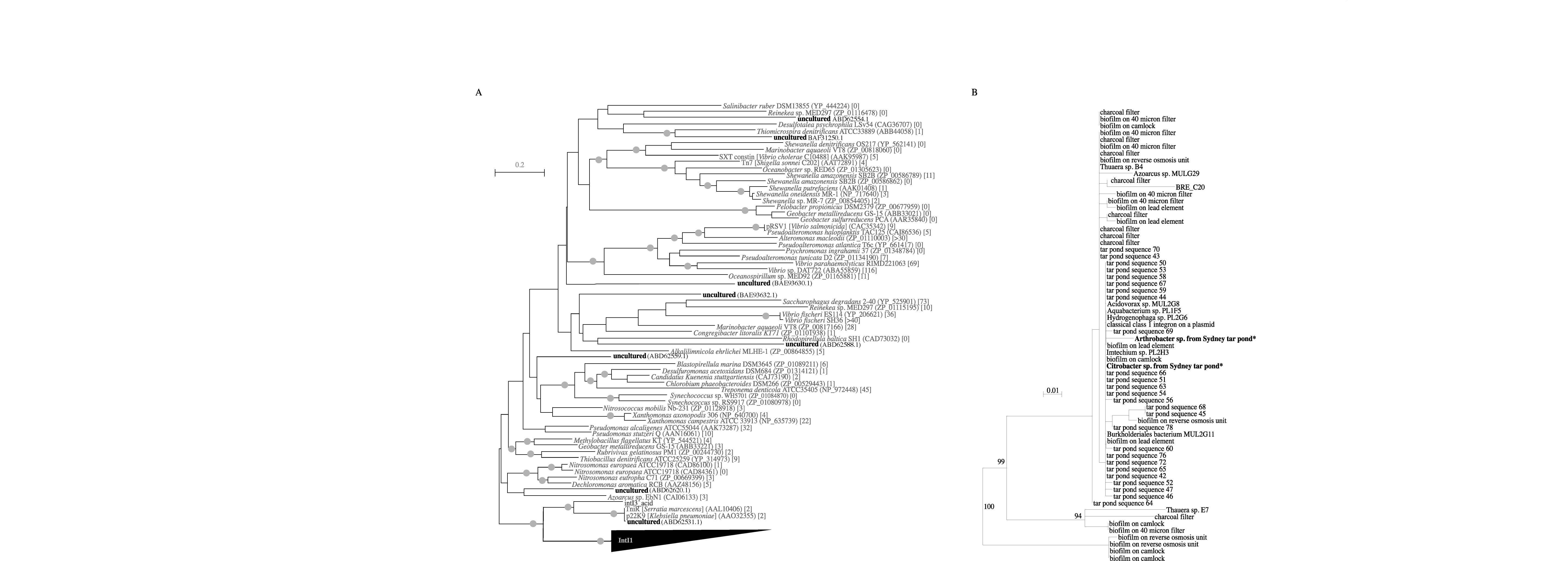

Supplement: Figure S4 — Integron integrase diversity in the Sydney Tar Ponds. (A) General IntI diversity. A diverse selection of IntI protein sequences were retrieved from NCBI for phylogenetic analysis, among these are eight from a deep-sea vent environmental survey of integrons illustrated in bold-type [23]. The number of cassettes in the associated array is indicated in square brackets where data is available. All integrase genes amplified in this study cluster within the black clade that is made up of class 1-associated integrases. Grey circles represent greater than 80% bootstrap support. (B) Nucleotide phylogeny of diverse class 1 intI sequences. This analysis includes all intI sequences amplified from the Tar Ponds as well as those collected from diverse isolates in addition to sequences amplified in a recent environmental survey of class 1 integrases [49]. RA×ML phylogeny of these sequences resolved three well-supported clades indicated by the bootstrap values at their nodes. (0.91 MB TIF) [file pone.0005276.s004.tif]

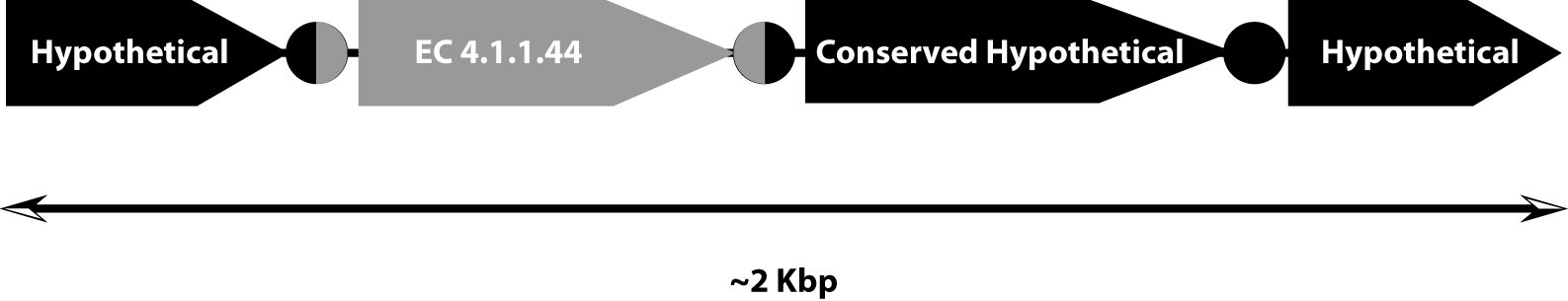

Supplement: Figure S5 — Partial integron gene cassette array obtained by long-walk PCR on DNA extracted from the Sydney Tar Ponds. (1.95 MB TIF) [file pone.0005276.s005.tif]

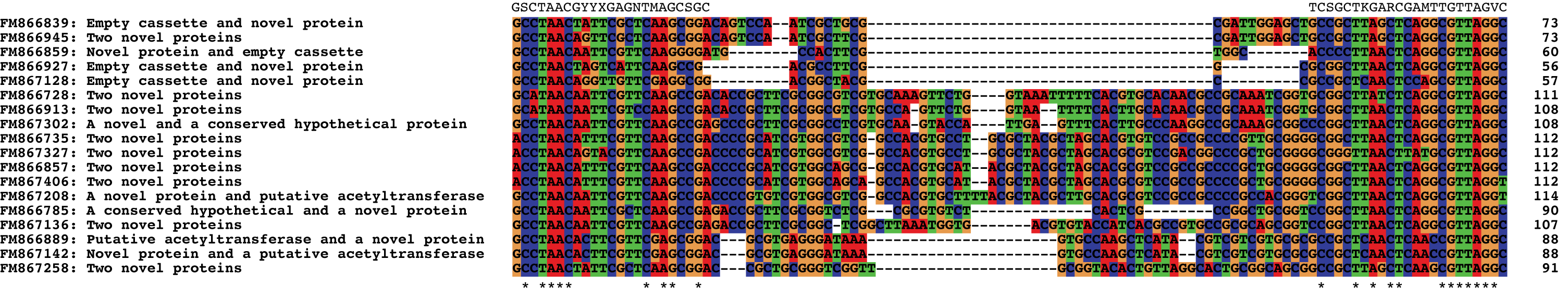

Supplement: Figure S6 — Nucleotide alignment of divergent attC recombination sequences embedded in multiple-cassette-amplicons. Primers used for cassette PCR are included at the top of the alignment [15]. None of the illustrated attC sequences would have been amplified with this primer pair. Asterisks represent positions in the alignment with identical nucleotides. Accession numbers and cassette-encoded functions are listed to the left of the alignment; putative functions are listed relative to their position (left or right) of the given attC. The sequence lengths of the attCs are indicated to the right. (0.69 MB TIF) [file pone.0005276.s006.tif]

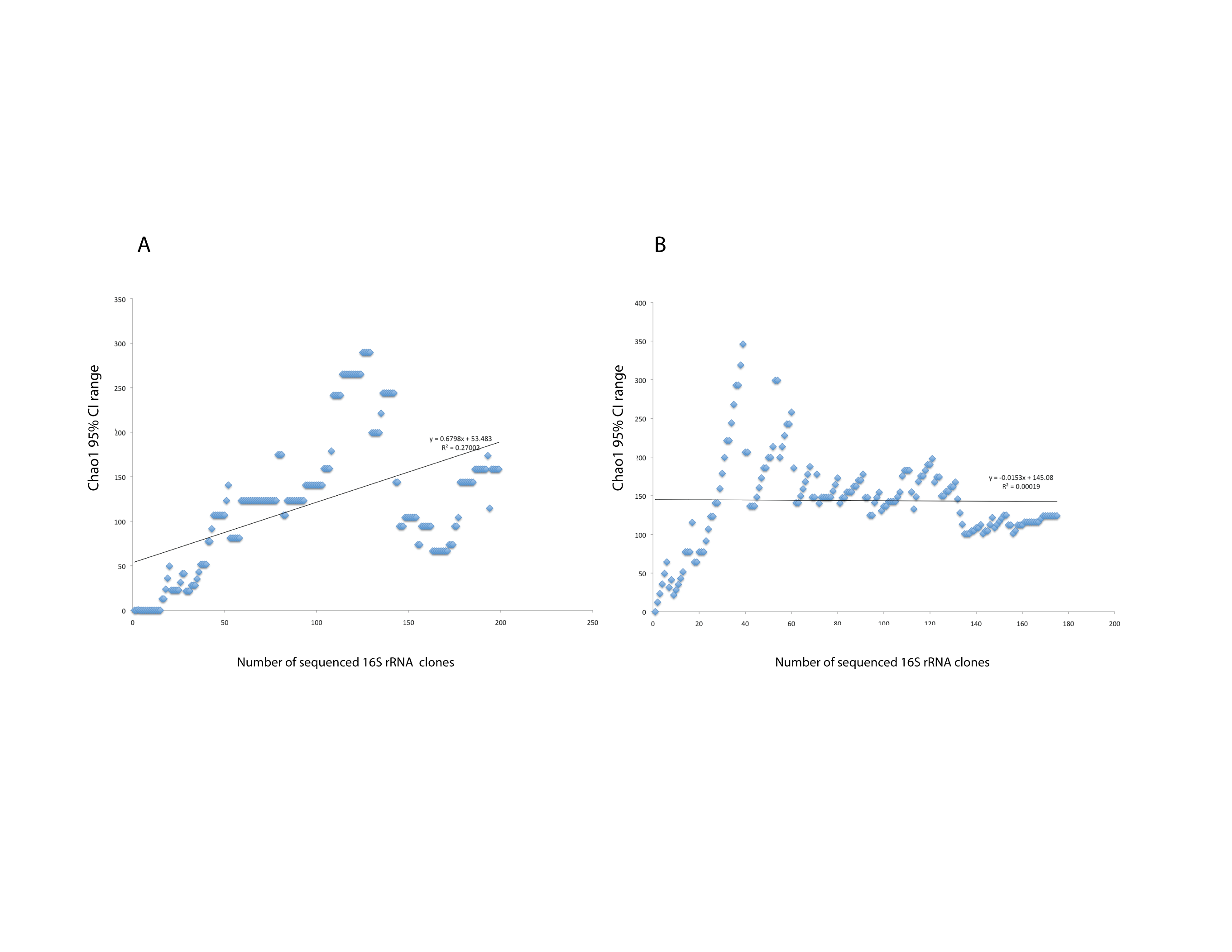

Supplement: Figure S7 — Dot plot of Chao1 95% CI range obtained from each ordered sampling incidence of 16S rRNA gene sequences amplified by either the archaeal-biased set (A) or the universal primer set (B). (0.59 MB TIF) [file pone.0005276.s007.tif]
