# Supplementary material for: Integron Gene Cassettes and Degradation of Compounds Associated with Industrial Waste: The Case of the Sydney Tar Ponds
Source: PLoS One. 2009 Apr 23;4(4):e5276. doi: 10.1371/journal.pone.0005276 (PMC2669170; doi:10.1371/journal.pone.0005276)
Supplement: Table S3 — (0.01 MB XLS) [file pone.0005276.s010.xls]

Supplementary Table 3. Contaminant levels within the Sydney Tar Ponds

Distribution of Contaminants in the Sydney Tar Ponds*

Contaminant (mg/kg surface soil)		Background	CCME**	Sydney Tar Ponds
**Petroleum hydrocarbons**				
	Benzene	0.3	5	580
	Toluene	0.64	0.8	370
	Ethylbenzene	0.085	20	71
	Xylene	0.55	17	290
**Polycyclic aromatic hydrocarbons**				
	1-Methylnaphthalene	0.35	NA	640
	2-Methylnaphthalene	0.37	NA	1,000
	Anthracene	0.66	NA	720
	Benzo(a)anthracene	1.4	10	380
	Benzo(b)fluoranthene	1.1	10	180
	Dibenzo(a)anthracene	0.2	10	29
	Chrysene	0.43	22	320
	Indenol(1,2,3)pyrene	0.9	10	100
	Naphthalene	0.4	22	3,200
	Pyrene	2.9	100	680

*Contaminant data collected from NS Department of Transportation and Public Works
**Canadian Council of Ministers for the Environment
Cassette-encoded genes implicated in downstream catabolites of contaminant
